# Towards a Holistic View of the Orchestration Between Sugar Transporters in Cereal Crops

**DOI:** 10.3390/plants15020201

**Published:** 2026-01-08

**Authors:** Xin’er Qin, Guoli Wang, Li Li, Yanbin Deng, Junli Chang, Yin Li, Xiangling Shen

**Affiliations:** 1 Key Laboratory of Three Gorges Regional Plant Genetics and Germplasm Enhancement, China Three Gorges University, Yichang 443002, China; 2 The International Science and Technology Cooperation Base (Genetic Engineering) of Chinese Ministry of Science and Technology, The Key Laboratory of Molecular Biophysics of Chinese Ministry of Education, College of Life Science and Technology, Huazhong University of Science & Technology, Wuhan 430074, China

**Keywords:** sugar transportation, sugar transporters, SWEET transporter, sucrose transporter, tonoplast monosaccharide transporter, AI-enabled protein analyses, multi-omics

## Abstract

Soluble sugars are the key photo-assimilates in higher plants, playing critical roles in growth, development, and stress regulation. The transport of sugars in plants involves the coordinated action between several sugar transporter families, including the SUT, STP, pGlcT, VGT, TMT, INT, PLT, SFP, and SWEET families. Over recent decades, numerous studies have elucidated the molecular functions of major sugar transporters. Phylogenetic and evolutionary analyses support the conservation of substrate specificity and transport direction, at least to some extent. Structural analyses have provided key insights into the structural–function relationships of important transporters (e.g., OsSWEET2b and AtSTP10), which can be effectively leveraged for artificial intelligence (AI)-enabled protein structure prediction and rational design. Advances in omics technologies now enable low-cost, routine transcriptome profiling and cutting-edge techniques (e.g., single-cell multi-omics and spatiotemporal RNA-seq), providing unprecedented ways to understand how sugar transporters function coordinately at multiple levels. Here, we describe the classification of major sugar transporters in plants and summarize established functional knowledge. We emphasize that recent groundbreaking advances in AI-enabled protein analyses and multi-omics will revolutionize molecular physiology in crops. Specifically, the integration of functional knowledge, AI-based protein analyses, and multi-omics will help unravel the orchestration of different sugar transporters, thereby enhancing our understanding of how sugar transportation and source–sink interactions contribute to crop development, yield formation, and beyond, ultimately boosting carbohydrate transport- related crop improvement.

## 1. Introduction

Sugars are important carbohydrates in plants and can be classified according to the number of structural units as monosaccharides (glucose and fructose), oligosaccharides (disaccharides are the most prevalent, such as sucrose and maltose), polysaccharides (starch, cellulose, etc.), bound sugars (glycolipids and glycoproteins, etc.), and sugar derivatives (sugar alcohols, etc.) [1]. The availability of sugars is coordinated with plant growth and development, and the levels of soluble sugars such as glucose and sucrose not only regulate the progression of plants from embryonic development to senescence and affect seed germination, seedling development, organ and tissue formation, but also orchestrate with light signals as signaling molecules to regulate certain metabolic pathways or gene expression [2,3,4,5]. Therefore, the transport and distribution of sugars play a crucial role not only in plant growth and development but also in the interaction with biotic and abiotic factors, and they are regulated via different carbohydrate transporters and metabolic pathways [6,7,8]. In line with this, sugar transporters have been identified as critical in the storage of various sugars in fruits and seeds, crop yield and quality, and plant–pathogen interactions [9,10,11,12,13].

To understand the biological functions and sugar transport mechanisms of sugar transporters and to foster new insights into sugar transporter manipulation and design, as well as sugar flux and carbon partitioning in crops, this study firstly summarizes the current knowledge about the functions, sugar transportation, and structural insights of three major sugar transporter groups (i.e., Sugar Will Eventually be Exported Transporter (SWEET) family, sucrose transporter (SUT) family, and the sugar porter (SP) family) present in the model plant species and major cereal crops (*Arabidopsis thaliana*, rice and maize). Then, we will briefly introduce recent advancements in multi-omics technologies and artificial intelligence (AI)-driven protein structure prediction and design, which have been proven to be applicable for enhancing the oil content of soybean [14]. In particular, we emphasize here that tools for AI-enabled protein structural analysis, prediction, and design have been rapidly developed and advanced. Comprehensive reviews on this topic have been published elsewhere [15,16]. While it has been acknowledged that AI can be used to enhance numerous fields of new biotechnologies, AI-designed proteins also demonstrate high stability, modularity, and engineering amenability, thus making AI-aided protein design a promising and powerful tool for crop improvement [17,18]. Promisingly, these new technologies can facilitate a holistic view of the orchestration between a set of sugar transporters for a given biological process critical for agronomic traits (for example, the grain filling of cereal crops). In addition, our study does not aim to comprehensively summarize the functional insights and molecular regulatory aspects of sugar transporters in plants, as some excellent systematic reviews or mini-reviews have already effectively covered these fields [19,20,21,22,23].

## 2. Sugar Transportation

### 2.1. Brief Introduction of Sugar Transportation and Transporters

Sucrose is formed via the dehydration and condensation of one molecule of the hemiacetal hydroxyl group of glucose with one molecule of the hemiacetal hydroxyl group of fructose. Sucrose is a highly stable non-reducing sugar, and a limited number of enzymes catalyze sucrose degradation in higher plants. These properties make sucrose suitable for long-distance transport and long-term storage in the phloem [24]. In most annual herbaceous plants (including many crops such as rice, maize, and wheat), sucrose is the main product of photosynthesis in the source leaves and the main form of carbohydrate for long-distance transport through the phloem for distribution to sink organs such as roots, stems, flowers, and seeds that can utilize or store assimilated products [20]. There are two main pathways for sucrose loading and unloading in the phloem: the symplastic pathway and the apoplastic pathway. In the symplastic pathway, sucrose synthesized by leaf photosynthetic cells enters the sieve element (SE) via plasmodesmata (PD) down a concentration gradient, and it is subsequently transported from the SE to companion cells (CCs) and parenchyma storage (P) sequentially via plasmodesmata, sucrose is transported according to the concentration gradient, and this process does not cross the cell membrane [25,26]. When sucrose is transported to the CC, it is converted to raffinose and stachyose. The raffinose family oligosaccharides (RFOs) are larger than sucrose and cannot diffuse back to the chloroplast through the PD but can be transferred to the sieve cells through large PD, thus accumulating in the phloem to maintain high sugar concentrations; this mechanism is known as polymer trapping [27,28]. After sucrose is transported directly to the sink cells via PD, it can be transported from the cytoplasm to the vacuoles for storage as sucrose or hydrolyzed by vacuolar invertase (VI) to produce glucose and fructose [29]. Sucrose synthase and cytoplasmic invertase (CI) in the cytoplasm can also convert sucrose to uridine diphosphate glucose (UDPG) and glucose and fructose, respectively. Following this, glucose can be metabolized to glucose-6-phosphate, which can be transported to the plastid and subsequently converted to starch [30,31]. Unlike the plasmodesmata-dependent symplastic pathway, the apoplastic pathway requires energy supply and the assistance of transmembrane transport carriers. Sucrose released from the vacuole to the cytoplasm is transported from the chloroplast to the phloem parenchyma (PP) at first, actively transported from PP to CC under the action of sugar transporter proteins to complete the loading of the phloem, and diffuses to SE via PD. After undergoing long-distance transport, sucrose in SE is finally effluxed and absorbed by the sugar transporter proteins sequentially into the PP of the sink storage tissue for further metabolism [25,26].

A large number of studies have shown that sugar transporters play a key role in the carbohydrate metabolic network and carbohydrate partitioning, serving as one group of the core functional proteins required for sugar transport, distribution, and accumulation [22,32,33]. According to the number of transmembrane helices and the type of protein structural domains, transporters known for the transportation of various sugars fall into the Sugar Will Eventually be Exported Transporter (SWEET) family, sucrose transporter (SUT) family, and sugar porter (SP) family [21]. SWEET facilitates the bidirectional transport of glucose, sucrose, fructose, etc. [32,34]. The function of SUT is mainly to load sucrose into the vascular tissues and to absorb sucrose directly into sink organs such as seeds and flowers [19], which implies the presence of transporters that efflux sucrose into the cell wall space. In some of the literature, the SP family is also designated as the monosaccharide transporter (MST) family, and together with the SUT family, it belongs to the major facilitator superfamily (MFS). For clarification and consistency, the term “SP” instead of “MST” will be used hereafter. The SP family has more coding genes than those of the SUT and SWEET families [35]. SP is mainly responsible for the distribution and transport of hexoses and sugar alcohols in the plant. During phloem unloading, sucrose is broken down into glucose and fructose by cell wall binding acid invertase (CWI) in the apoplast and requires the involvement of SP to translocate monosaccharides from the apoplast space into the sink cells or their vacuoles for storage [25,36]. In the sink tissues of cereal crops (maize, sorghum, and sugarcane, etc.), *cytoplasmic invertases* (also known as the *alkaline-neutral invertase* (*INVAN*s)) are expressed, but their expression patterns do not directly correlate with the dynamic changes in sucrose or glucose, suggesting that complex control at the protein level affects cytosol sugar metabolism [37,38,39].

### 2.2. Sugar Will Eventually Be Exported Transporter (SWEET)

SWEET is a newly discovered family of sugar transporter proteins that are widely found in prokaryotes, eukaryotic unicellular organisms, higher plants, and animals [40,41]. In 2010, Chen [42] first identified and named SWEET as involved in glucose efflux in *Arabidopsis thaliana* and *Oryza sativa* using fluorescence resonance energy transfer sensor technology (FRET) and performed a preliminary study on their structure and function. At present, with the continuous innovation and development of genome sequencing technology and transcriptome research methods, the structural characteristics and physiological functions of SWEET are being studied more clearly.

#### 2.2.1. Structure and Evolution of SWEETs

**Figure 1:**
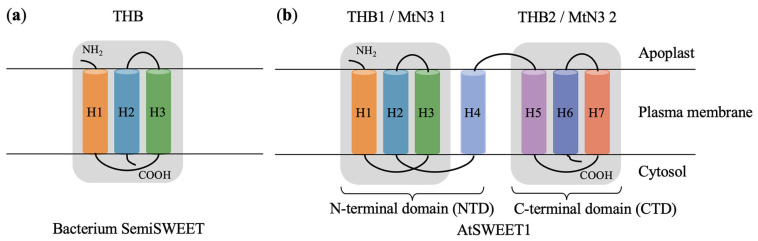
Membrane topology diagrams of bacterium SemiSWEET (**a**) and AtSWEET1 (**b**) localized in the plasma membrane. TM4 and THB1 constitute the N-terminal domain (NTD), while THB2 forms the C-terminal domain (CTD) of AtSWEET1. THB, triple-helix bundle. Typical SWEET proteins in higher plants (using the AtSWEET1 here as an example) have two THBs, THB1 and THB2, that contain the transmembrane helices 1–3 and 5–7 (TM 1–3, TM 5–7), respectively.

Unlike SUT and SP, both eukaryotic SWEET and bacterial SemiSWEET belong to the MtN3 saliva family (PFAM database code PF03083; see http://pfam.xfam.org, accessed on 1 November 2025), which are polytopic membrane proteins characterized by the MtN3/saliva domain. The amino acid sequence of MtN3/saliva domain is very close to the proline–glutamylamine loop (PQ-loop) of another member of the membrane protein family (PF04193); thus, SWEET can also be referred to as MtN3/PQ-loop/SWEET [43,44]. In eukaryotes, SWEET consists of seven transmembrane helices (TMHs), which contain two triple-helix bundles (THBs) formed by the MtN3/saliva domains (TM 1-3, TM 5-7) together with a linking TMH, and form a 3-1-3 structure [45] (Figure 1). Among the seven TMHs that constitute SWEET, TM4 mediates the critical contact between SWEET protomers and, due to poor conservation, acts as an inversion linker interacting closely with THB1 to form the N-terminal domain of SWEET, but it barely contacts THB2 to form the C-terminal domain within the protomer [46]. In addition, TM4 strongly binds with the first THB within the protomer region and thus arbitrates key contacts among other proteins to modulate gene expression [47]. Two THBs bind to each other in a reverse parallel manner to form a functional translocation pore complex. The reason why SWEET requires the formation of homo- or heterodimeric polymorphs, with the most likely scenario being dimerization, to generate translocation pores to achieve its transport function is that a single molecule is not sufficient to form a hydrophilic channel on a hydrophobic membrane that allows for the passage of molecules such as glucose [48]. The structural analysis has confirmed that OsSWEET2b forms homomeric trimers [46]. SemiSWEET in bacteria, on the other hand, is a small-molecular-weight protein or peptide composed of three TMHs containing one MtN3/saliva domain [48], which probably forms at least one dimer to form a translocation pore [44].

Sequence similarity networks, phylogenetic trees, and sequence alignments were analyzed for the presence of SWEET and SemiSWEET, and two mechanisms currently explain the evolutionary relationship between SWEET and SemiSWEET: (1) SWEET is generated via the replication and fusion of SemiSWEET; (2) SWEET arises from the fusion of SemiSWEET in bacteria and archaea. In conclusion, gene duplication and fusion are key factors driving the evolution of SWEET [41,47,48].

#### 2.2.2. Sugar Transportation Functions of SWEETs

***Structural insights into SWEET molecular functions***. The SWEET family is a class of passive transporters that transport substrate molecules via facilitated diffusion and can be localized in different cellular regions such as the tonoplast (e.g., AtSWEET16 and 17) [49], the Golgi apparatus (e.g., AtSWEET9 and 15) [50] and the plasma membrane (e.g., AtSWEET1, 8, 9, 11, 12, and 15) [51,52]. SWEET functions as a transmembrane transporter between different cells or organs, relying on its conformational changes to enable substrate binding on one side of the membrane and translocation and release on the other side [41,51,53]. Cheng and Selvam [50,52] combined extensive molecular dynamics (MD) simulations and Markov state models (MSMs) to study the conformational dynamics and transport mechanism of OsSWEET2 in rice for transporting glucose across the membrane and described this mechanism as an alternate access transport mechanism [54]. SWEET has three different conformational states during transport: the inward-open state (IF), the occluded state (OC), and the outward-open state (OF): the cavity of the SWEET faces the extracellular side in the OF state, the SWEET in the OC state has both ends closed, and IF state cavity shifts towards the cytoplasmic side of the plasma membrane [55,56,57]. The formation of hydrogen bonds between amino acid residues leads to the rearrangement of the hydrogen bonding network and the proximity of the helices to each other, resulting in the partial closure of the transporter protein and the transformation of the conformational crystal structure or stabilization in an intermediate state. MD simulations take OsSWEET2 of glucose unbound (apo) in the IF state as a starting point, and the characterization of apo via transition path theory (TPT) illustrates that the transition from IF to OF conformation passes through the following six stages of intermediate states: extended IF, IF, partial IF-OC, OC, partial OC-OF, and OF. The binding of glucose reduces the free energy barrier of the intermediate state transition to increase the selectivity of the transport protein for the substrate and the rate of transport [50,52,54,55,56,57].

***Phylogenetic clades of SWEET proteins associated with substrate specificities***. In Arabidopsis and rice, for example, the SWEET family is divided into four branches: Clades 1, 2, 3, and 4. SWEETs belonging to the same branch do not represent participation in the same physiological processes, but selection for transporting monosaccharides or disaccharides is relatively consistent [47,58,59]. Clade 1 (e.g., OsSWEET2b, AtSWEET1-3) mainly transports 2-deoxyglucose. Clade 2 (e.g., AtSWEET4-8) mainly transports glucose. Clade 3 (e.g., AtSWEET9-15) preferentially transports sucrose across the plasma membrane. Clade 4 (e.g., AtSWEET16-17) localizes to the tonoplast, mainly mediating the unidirectional transport of fructose [58,59,60]. Based on the transport of mono- or disaccharides by SWEET localized in different regions of the cell, SWEET has several physiological functions such as apoplast loading or unloading in phloem, seed germination, pollen nutrition, nectar secretion, regulation of sugar distribution, and senescence of plant tissues. Currently, some members of the SWEET family have been identified and studied in Arabidopsis, rice, sugarcane, maize, and sorghum [26,53,61,62,63,64,65,66,67,68,69,70,71].

***Expression analyses of SWEET-encoding genes in Arabidopsis and cereal crops***. The distribution and metabolism of carbohydrates in sugarcane is a complex genetic regulatory process that relies on the proper functioning of a sugar translocation network, a carbon assimilation network centered on photosynthesis, and a carbohydrate metabolic network dominated by the synthesis and degradation of sugars. Hu et al. [26] compared the transcriptome expression profiles of *Saccharum spontaneum* and *Saccharum officinarum* leaves and stems at different developmental stages via RNA-seq analysis, finding that *SWEET1a/2a/4a/4b/13a/16b* were the candidate genes responsible for sugar differences between the sugarcane species mentioned above. In addition, based on transcriptome data from leaf gradient segments, *SWEET4b/11a* was expressed at the leaf bases (sink tissue), and *SWEET13a/13b/13c* was expressed mainly in the maturing and mature zones of the leaf. *AtSWEET11* and *AtSWEET12*, homologs of *SWEET13a/13b/13c* in Arabidopsis [44], belong to the Clade 3 subfamily, and the expressed proteins are present on the plasma membrane of parenchyma cells in the phloem, which are low-affinity/high-transport-capacity transporters of sucrose and are involved in the transport of sucrose to the apoplast. Chen et al. [61] constructed the *atsweet11;12* double mutant; the rosette leaves and roots exhibited growth defects and accumulated high levels of hexose and starch in the mature leaves, indicating reduced translocation of leaf photosynthetic assimilation products to sink organs such as roots. These results implied that *SWEET13a/13b/13c* and *AtSWEET11/12* mediated sucrose efflux to leaf apoplasts, and *SWEET4b/11a* is associated with sucrose efflux from leaves to stems. In sugarcane stems, *SWEET13c* was expressed in both maturing and mature stems, whereas the Clade-1 *SWEET2a* was mainly expressed in immature stems, suggesting that *SWEET13c* may be involved in sucrose accumulation in mature stem cells, whereas *SWEET2a* is involved in sucrose efflux from source to sink tissues [26]. *SWEET13c* showed a circadian expression pattern, and to further verify the role of *SWEET13c* in sugar transport, Hua et al. [32] overexpressed *SsSWEET13c* in Arabidopsis and found that leaf sugar content significantly decreased, while root length/weight, stem weight and fresh weight significantly increased, which may be due to *SsSWEET13c*-drived leaf sugar efflux and transported to roots and stems, suggesting that *SsSWEET13c* is a key candidate gene involved in sugar flux and crop carbon allocation. *SbSWEET13a/13b/13c* genes in sorghum are also the most highly expressed of the Clade 3 subfamily genes in the leaf phloem and stem, and they play an important role in sucrose accumulation in sorghum stems [39].

***Representative genetic and functional studies of SWEETs in Arabidopsis and cereal crops***. OsSWEET11, a homologous protein of AtSWEET11/12 in rice, which belongs to the Clade 3 subfamily, was expressed in the leaf phloem and was not only associated with sucrose transport [26] but also involved in reproductive processes such as pollen development and seed filling [62,63]. Chu et al. [62] found that *OsSWEET11* was highly expressed in spikes and anthers, mutations caused a reduction in pollen starch content, and the accumulation of *OsSWEET11* transcripts in the wild-type overlapped with the timing of pollen abortion in mutants, suggesting that OsSWEET11 is required for pollen development. In maize, grain filling is the process via which sucrose is first hydrolyzed to glucose and fructose and then enters the endosperm through the monosaccharide transporter of the basal endosperm transfer layer (BETL), and it has been recently found that sucrose can enter the endosperm via BETL directly mediated by ZmSUGCAR1 without hydrolysis in maize [64]. An analysis of the expression profiles of two RNA-seq datasets, soybean whole seed at eleven stages of reproductive tissue development and three vegetative tissues, revealed that the overall expression of *SWEET*s gradually increased during seed filling in soybean and then decreased as seed maturity increased [65]. In addition to *OsSWEET11*, Clade 3 members *OsSWEET15* and *OsSWEET4* were preferentially expressed in the glumes, and their mRNA levels reached higher levels at later stages of glume development [65,67]. Yang et al. [63] obtained an *ossweet11;15* double mutant, where the glumes did not form a functional endosperm and the pericarp starch accumulated to higher concentrations; however, no significant amount of starch grains was found in the endosperm. The *OsSWEET11* mutant exhibited defective endosperm and slightly delayed degeneration of nucellar cells, and the above results suggest that OsSWEET11 and OsSWEET15 are required for sucrose efflux and contribute to sucrose transfer to the nucellar epidermis/aleurone interface.

Maize seeds and stalks are rich in carbohydrate resources, and the molecular regulatory mechanisms of sugar transport in maize seeds have been revealed. Shen et al. [66] resolved the high-resolution spatiotemporal transcriptional profiles of the *STP*, *SUT*, and *SWEET* family genes in maize seeds and then proposed a molecular mechanism model for sugar transport. Among them, the Clade 3 member ZmSWEET11/13b transports sucrose from the PC to the apoplast space at the maternal-offspring junction, and subsequently BETL takes up the sugar in the apoplast space through two pathways: (1) sucrose uptake driven by the sucrose transporters ZmSUT1 and ZmSWEET11/13a; (2) hydrolysis of sucrose to monosaccharides by CWI, followed by the monosaccharide transporter protein ZmSTP3, Clade 1 member ZmSWEET3a and Clade 2 member ZmSWEET4c driving the uptake of sucrose as monosaccharides. Meanwhile, the Clade 3 members ZmSWEET14a/15a and ZmSUT4 are responsible for the translocation of sucrose from the peri-embryo region (endosperm) to the embryo, respectively. *ZmSWEET4c* is expressed in BETL and is required for seed germination and the differentiation of the endosperm transfer layer [10]. ZmSWEET13s (a, b, c) are homologous SsSWEET13c proteins and belong to the Clade 3, for which the gene expression level is the highest in the leaf vasculature. Bezrutczyk et al. [67] obtained triple knockout mutants of *ZmSWEET13a/b/c* by the genome-editing technique, where mutants had impaired photosynthesis, accumulated large quantities of soluble sugars and starch in leaves, and had severely stunted plants. Furthermore, RNA-seq data showed that the mRNA levels of several genes encoding light-harvesting complex and chlorophyll/tetrapyrrole biosynthesis were significantly reduced, and transcripts related to starch biosynthesis and sucrose degradation were also affected, which could indicate that ZmSWEET13s play a major role in maintaining normal photosynthesis, as well as in sucrose uptake and translocation in the apoplasts of plants. Wang et al. [68] identified a *cst1* mutant in the EMS mutant population, and after map cloning and CRISPR/Cas9 validation, demonstrated that the *cst1* gene encodes the SWEET Clade-1 subfamily glucose transporter protein CST1. On one hand, the mutant exhibited stomatal closure, chlorophyll degradation, and insufficient seed filling; on the other hand, according to transcriptomics and metabolomics data, senescence-associated genes (SAGs) were significantly up-regulated, genes involved in sucrose and starch synthesis were significantly downregulated, and genes responsible for sucrose and starch degradation were significantly upregulated, resulting in a significant reduction in starch and most of sugars in the mutants, suggesting that CST1 regulates stomatal opening, photosynthesis, and carbohydrate accumulation. Plant tissue senescence is closely related to sugar accumulation [69]. The Clade 3 *AtSWEET15* (*SAG29*) was highly induced to be expressed during leaf senescence, and its transcripts accumulated gradually with leaf senescence [70]. OsSWEET5 is a member of the Clade 2 subfamily that transports galactose, and its overexpression leads to stunted growth and early failure in rice [72].

Based on the established knowledge of SWEET proteins, we suggest that integrating the detailed spatiotemporal expression profiles, phylogenetic clades, subcellular localization, and biochemical characterization will greatly help us to understand the functions and biological roles of SWEET proteins in a given plant species, including their sugar substrates and coordination in different tissues to affect organ development.

### 2.3. Sucrose Transporter (SUT)

SUT is a class of transmembrane proteins with sucrose transport activity in plants that can regulate the transport and distribution of sucrose during plant growth, and the transport process relies on H^+^-ATPase to form a plasma membrane electrochemical potential difference; thus, they are also named sucrose/H^+^ co-transporters (SUCs) as well [19]. In 1992, Riesmeier et al. [73] identified the first sucrose transporter family gene, SoSUT1, in the dicotyledonous plant *Spinacia oleracea* and hypothesized that the gene functions as a sucrose transporter. Subsequently, *SUT* family genes have been continuously cloned in monocotyledons (e.g., sugarcane, wheat, barley, sorghum, rice, maize) and dicotyledons (e.g., potato, Arabidopsis) [74,75,76,77,78]. Furthermore, the protein structure, classification, evolution, and physiological functions of SUTs have become more in-depth and abundant.

#### 2.3.1. Structural Knowledge of SUTs

**Figure 2:**
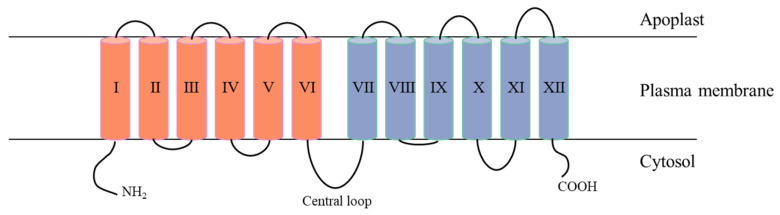
A membrane topology diagram of AtSUC1 localized in the plasma membrane. The transmembrane helices (numbered in Roman numbers) are shown in different colors. Identical colors were used for the respective transmembrane helices of the first and the second halves. There is a central loop structure between TMH VI and VII, which has no effect on sucrose transport activity.

SUT belongs to both the major facilitator superfamily (MFS) and glycoside–pentoside–hexuronide (GPH) cation symporter family [73]. The amino acid sequence and protein structure of SUT are relatively conserved, and its typical structure contains twelve transmembrane α-helices with both the N and C termini located on the cytoplasmic side of the membrane [24,79] (Figure 2). It has been shown that differences in amino acids in the N-terminal structural domain could determine the magnitude of SUT affinity for sucrose to alter its transport activity [80], as well as that the N-terminal structural domain may be related to the localization of SUT, with both SbSUT4 and SsSUT4 containing a segment of the LXXLL motif that may target SUT to the tonoplast [14,72]. Sauer et al. [24] proposed a model for the arrangement of TMH on the plasma or tonoplast, where TMH I, IV, VII, and X form an hourglass-like structure surrounded by the remaining eight helices to form a substrate pore. According to current studies, SUT may form functional dimers with homotypic proteins or other cellular components in a redox-dependent manner; for example, two SUT1 subunits in tomato can form homodimers, SUT1 plus SUT4 can form heterodimers, and SUT1 plus SUT2 can form heterodimers with lower affinity than homodimers [81,82].

#### 2.3.2. Phylogenetic Insights into SUTs

Based on homology analysis and cluster analysis of SUT encoding genes and amino acid sequences, SUT can be classified into five subclades: SUT1, SUT2, SUT3, SUT4, and SUT5. The SUT1 subclade is found only in dicotyledons, the SUT2 and SUT4 subclades are found in mono- and dicotyledons, and the SUT3 and SUT5 subclades are unique to monocotyledons [40]. Zhang et al. [83] clustered 78 SUTs from eight dicotyledonous and ten monocotyledonous species based on amino acid sequences, and the evolutionary tree results indicated that SUT1 and SUT4 originated from the same branch while SUT2, SUT3, and SUT5 originated from another branch. Similarly, the hypothesis proposed by Reinders [84] suggested that SUT2 and SUT4 are the common ancestors of SUTs in mono- and dicotyledonous species, predicting that after the differentiation of monocots and dicots, SUT4 evolved to form the dicot-specific SUT1, and SUT2 formed the monocot-specific SUT3 plus SUT5. SUT1 is the subclade with the highest sucrose affinity, and it is mainly expressed in guard cells and the plasma membrane of sieve elements (SEs) in apoplasts [14]. The SUT2 member in dicots is localized to the plasma membrane and, from the protein structure, has a central loop structure between TMH VI and VII with a length of about 60 amino acids, which has no effect on sucrose transport activity [66,73]. SUT3 has the highest specificity and moderate affinity for sucrose and is localized to the plasma membrane of SEs or companion cells (CCs) [85,86]. SUT4, localized on the tonoplast or plasma membrane in different species, whose affinity for sucrose is about one-tenth that of SUT1, has a specificity for transporting substrates that are intermediate between SUT1 and SUT3 and belongs to the group of low-affinity/high-capacity transporters [87,88,89]. SUT5 members are not significantly characterized. Compared with OsSUT1, a member of the SUT3 subclade in rice, OsSUT3, belonging to the SUT5 subclade, has a higher affinity for sucrose, reduced sensitivity to pH during transport, and broader substrate specificity [90].

#### 2.3.3. Sugar Transport Functions of SUTs

As mentioned earlier, sucrose is loaded into the phloem after synthesis in the chloroplasts, and the process of sucrose excretion from PP via the apoplast pathway and flow into CC requires the assistance of SWEET plus SUT. Sucrose is excreted from SE/CC during phloem unloading and is taken up into the sink parenchyma cells with the transport of SUT [25]. Consequently, SUT has an important effect on the long-distance transport of sucrose from source to sink, and understanding the physiological functions of different subclades of SUT in different plants can help us to elucidate the mechanisms of carbohydrate partitioning and transport in plants.

***The functions of SUTs in rice, maize, and sorghum***. The sorghum variety BTx623 belongs to a grain sorghum type that mainly stores the photo-assimilates as starch in the seed; in contrast, the sweet sorghum Rio accumulates sucrose in the vacuoles, cytoplasm, and extracellular spaces of storage parenchyma cells. From flowering to maturity, sweet sorghum stems can accumulate large amounts of soluble sugars (mainly sucrose) and starch, totaling up to three times that of BTx623 [91,92]. Milne et al. [74] cloned six *SUT*s in Rio and BTx623, respectively, and complementation experiments in the yeast mutant strain SEY6210 (unable to grow with sucrose as the sole carbon source) demonstrated the ability of SUTs to transport sucrose. More specifically, the gene expression results indicated that *SbSUT2* and *SbSUT5* were most strongly expressed in the internodes and may be crucial for apoplast unloading, the recycling of sucrose leaked from the apoplast, or the apoplast loading of sucrose recycled from the stems. *SbSUT1*, *SbSUT4*, and *SbSUT6* were highly expressed in mature source leaf tissues and are potential candidates for phloem loading in apoplasts, with *SbSUT1* and *SbSUT6* having higher expression in BTx623, which is consistent with the finding that *SsSUT1* was less expressed in all tissue types of sugarcane high-sugar species than in low-sugar species [75]. ZmSUT1 was previously thought to be associated with sucrose loading into the phloem, as it is notably expressed in mature leaves and in CC [76]. Slewinski et al. [93,94] isolated and characterized the maize mutant *sut1* with excessive carbohydrate accumulation in mature leaves, guttation fluids secreted by special leaf structures hydathodes containing more than 700 mg/g of sugar and not easily evaporated, the negative feedback regulation of photosynthesis via soluble sugar accumulation, decreased stomatal conductance that manifested by leaf greening and early senescence, and reduced carbohydrate delivery to sink tissues leading to inhibition of reproductive development and defective nutritional growth, identical to the phenotype of WT treated with cold-girdle and resulting in blocked phloem transport. In summary, ZmSUT1 plays a vital role in loading sucrose into the leaf phloem and exporting it to the sink tissue. Compared to *ZmSUT1*, *OsSUT1* was expressed at lower levels in source leaves, and the source leaves of anti-sense suppression lines showed no significant differences in photosynthesis, leaf carbohydrate content, and phloem loading compared to WT, while seed filling was significantly reduced, indicating that OsSUT1 may not play a dominant role in rice phloem loading [95,96]. Equally important, the RT-PCR-based gene expression results showed that *OsSUT1* gradually returned to an almost undetectable level after peak expression at 5–7 days after anthesis [97] and that *OsSUT1* was expressed in the cell plasma membrane of the aleurone layer around the endosperm [98], suggesting that OsSUT1 is involved in the transport of assimilates across the aleurone layer to the developing seeds. OsSUT2, which belongs to the same branch as ZmSUT2 and SbSUT4 for amino acid homology up to 91%, localized to the tonoplast, is expressed in the mesophyll and bundle sheath cells, and it is thought to transfer sucrose from the vacuole cavity to the cytoplasm [20,87,99,100]. When *OsSUT2* was disrupted, mutant plants were reported to exhibit decreased tiller numbers and seed weight, as well as increased fructose, sucrose, and glucose contents, together with decreased contents of exported sugars in mature leaves, indicating that OsSUT2 can affect the movement of sucrose to the phloem [68]. The phenotype of *zmsut2* homozygous mutant plants is highly similar to that of *ossut2* mutant plants, but Leach et al. [100] attributed this similarity to the inability of sucrose to be exported from the vacuoles in photosynthetic cells rather than the inability to load sucrose into the SE-CCs of phloem.

***The functions of SUTs in sugarcane***. Sugarcane is an important model crop for studying SUT, and sucrose accumulated in the stalk can exceed 50% of the dry weight (DW) [101]. Zhang et al. [75] identified and named six *SUT* genes from *S. spontaneum* (designated as *SsSUT1* to *SsSUT6*). *SsSUT1* was expressed at a higher level in the source tissue (leaves) than in the sink tissue (stems) in seedling plants, with the opposite trend noted in mature plants. *SsSUT4* was expressed at similar levels in seedling and mature plant tissues, suggesting a correlation between *SsSUT1/4* and sugar content differences among sugarcane species. *SsSUT5* and *SsSUT6* were highly expressed in source leaves, and their products contributed to phloem loading. In a study by Rae et al. [102], the sugarcane *ShSUT1* was most strongly expressed in regions with high sucrose flux, especially in mature leaves that export sucrose and in stem internodes that accumulate sucrose. Next in importance, the results of immunolocalization experiments indicated that ShSUT1 was not present in the leaf vascular bundle phloem, probably making it similar to OsSUT1, which is not involved in leaf phloem loading [95,96]. Glassop et al. [103] used RNAi technology to significantly reduce *ShSUT1* expression by 85–92% in stem internode 7, but there was little difference in the stem sucrose concentration between transgenic lines, possibly implying that unloading of the stem phloem relies more on the symplastic pathway than the transporter and that its encoded protein may contribute to the partitioning of sucrose between the transporter and storage tissues, consistent with the previous findings [102].

***Functional comparison of SUTs between dicotyledons and monocotyledons***. The function of SUT1 in dicotyledons and monocotyledons is quite different, with *ShSUT1* and *OsSUT1* in monocotyledons being less expressed in the source leaves and probably not having a dominant effect during phloem loading. AtSUC3 in dicotyledon plants has similar sequence and kinetic characteristics to ShSUT1 [80], and *AtSUC2* is strongly expressed in both the collection phloem and the transport phloem and plays a role in sucrose loading, mainly in the phloem. The promoter *CmGAS1p* is expressed only in the small leaf veins of mature leaves (i.e., the collection phloem) but not in the large leaf veins and the vascular tissue of the rootstock (i.e., the release phloem and the transport phloem). Srivastava et al. [77] expressed *AtSUC2* cDNA from *CmGAS1p* in the *atsuc2* mutant background, and if AtSUC2 was involved in the sucrose efflux of transport phloem, sucrose along transport phloem was not lost to the outer tissues after gene deletion, resulting in a reduction in carbohydrate levels in the petiole. However, *AtSUC2* was not expressed in the transport phloem, and the whole leaves of the plant (including leaves and petioles) accumulated large amounts of sugars and starch. The results mentioned above suggest that AtSUC2 did not play a major part in transport phloem efflux. *AtSUC2* knockout plants with the *Atsuc2-4* allele with T-DNA insertion in the second intron still yielded living seeds, suggesting that phloem transport continued in the absence of AtSUC2 [78].

### 2.4. Sugar Porter (SP) Family

SPs are widely present in prokaryotes and eukaryotes and are mainly responsible for the selective transport of monosaccharides and sugar alcohols in plants. The systematic identification of *SP* genes in *Arabidopsis thaliana* was carried out in 2007 [104,105,106]. Based on phylogenetic and conserved structural domain analysis, SP can be divided into seven subfamilies [104,107]: STP (sugar transporter protein), PLT (polyol/monosaccharide transporter), VGT (vacuolar glucose transporter), INT (inositol transporter), pGlcT/SGB1 (plastidic glucose transporter/suppressor of G protein beta1), TMT (tonoplast monosaccharide transporter), and ERD6-like/SFP (early response to dehydration-like/sugar facilitator protein, ERDL/SFP).

#### 2.4.1. Structures of Sugar Porter Proteins

**Figure 3:**
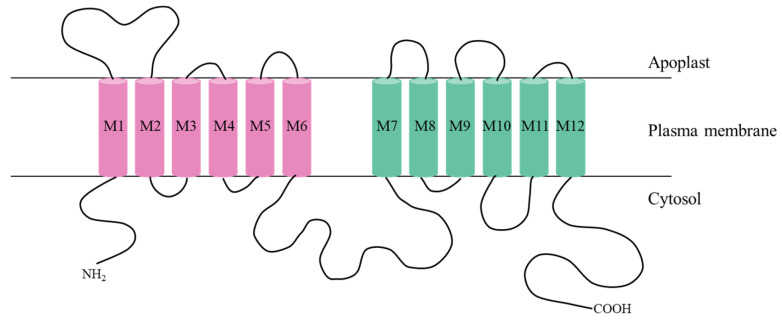
A membrane topology diagram of AtSTP10 localized in the plasma membrane. The transmembrane helices are shown in different colors. Identical colors were used for the respective transmembrane helices of the first and the second halves.

SPs contain the same conserved structural domain Sugar_tr, except for INT and TMT, which each have a Sugar_tr domain at their N-terminal and C-terminal ends; all other SPs contain only one Sugar_tr structural domain [108]. The number and distribution of TMHs in the SP family have a tendency to diversify, with most containing 12 transmembrane-spanning domains. For instance, the number of THMs in SbSTP (Figure 3) and SbPLT is mainly concentrated around 10, 11, and 12, while a few members contain 7, 8, and 9 TMHs [108]. SPs are integral membrane proteins that interact to form a central pore, thereby allowing soluble monosaccharides to span hydrophobic membranes [106]. TMT has a very long hydrophilic central loop consisting of about 320 amino acids, which can connect transmembrane structural domains 6 and 7 [108,109]. Bavnhøj et al. [110] established a molecular dynamics model for glucose transport by resolving the crystal structure of STP10. When STP10 is in the outward open conformation, protons and glucose substrates enter the central binding site of the glucose transport protein, forming a high-glucose-affinity state. As the structural domain moves, STP10 gradually transitions to the inward open state, the affinity for glucose binding decreases until glucose is released and the deprotonated conformation is formed, and finally, it returns to a more stable outward open state (Figure 3).

#### 2.4.2. Sugar Transport Functions of MSTs

***Substrate specificity of SP members from different subfamilies***. Existing studies have shown that STP, PLT, TMT, and VGT are high-affinity monosaccharide/H^+^ transport proteins [20,110,111,112] and that different MST subfamilies have different degrees of substrate specificity during sugar transportation [113]. Most STPs are localized to the plasma membrane and have various substrate-binding properties; for instance, OsSTP4 and OsSTP6 can transport hexoses such as glucose, fructose, galactose, and mannose [114,115]. AtSTP1, AtSTP2, AtSTP3, AtSTP4, AtSTP6, and AtSTP11 have different affinities for glucose, xylose, mannose, and galactose, but they cannot transport fructose [111]. A few STPs can only transport specific monosaccharides, such as AtSTP9 and AtSTP14, which exclusively transport glucose and galactose, respectively [116,117]. PLT is localized at the plasma membrane and can transport hexoses and polyols; for example, AtPLT5 mediates H^+^-Symport with myo-inositol, glycerol, and ribose [116]. The transformed strains of *SbPLT5* and *SbPLT6* were grown in fructose-added deficient medium, indicating that *SbPLT5* and *SbPLT6* can transport fructose [107]. Members of INT, localized to the plasma membrane or vacuole, can transport inositol (including epimers and derivatives) [113,118], and SbINT3 can transport glucose and mannitol in yeast [107]. Members of the TMT and VGT subfamily of proteins are localized to the tonoplast, and VGTs are H^+^/glucose anti-transport proteins such as AtVGT1 and AtVGT2 [119]. TMT actively transports glucose and fructose into the vacuole, and AtTMT1/2 also possesses sucrose transport activity [120,121]. pGlcT, such as SbpGlcT1, transports glucose. The EDR6-like subfamily may be passive (but selective) facilitative transporters for hexoses such as SbERD3, AtERD6, and AtESL1 (EDR6-like 1) [107,119].

***Spatiotemporal expression patterns of TMTs and the related functions***. Plant vacuoles account for 90% of the cell volume, and the tonoplast separates the contents, such as various sugars, polyols, organic acids, amino acids, and ions, from the surrounding cellular components; thus, there are various transporters, channels, and pumps on the tonoplast [119,122]. More specifically, the strong proton-motive force (PMF) of the tonoplast is maintained by three proton pumps. Sucrose and its catabolic products, glucose and fructose, are actively transported into vacuole storage mediated by TMT/VGT [20,108]. There are three *VGT* gene copies in Arabidopsis, among which *AtVGT1* is almost exclusively expressed in anthers, and knockdown causes delayed flowering and a significant reduction in seed germination rates [119]. In several crop species, *TMT* and *VGT* expression is closely related to sugar transport and accumulation in the root and stem. *SbTMT1* and *SbTMT2* are strongly expressed in leaves and mature internodes from booting to flowering, and both are significantly more expressed in sweet sorghum stems than in seed sorghum stems, indicating that *SbTMT1/2* is a candidate gene for the control of sucrose accumulation in sweet sorghum stems [120]. The number of *TMT* alleles in sugarcane increased compared to sorghum, with relatively high expression of *SoTMT2a* and *SoTMT2b* in internode 3 and intermediate internode 9, which are at maturity or early maturity, respectively, while *SoTMT1* increased the expression level with increasing internode maturity [123]; thus, it can be concluded that *SoTMT1*, *SoTMT2a* and *SoTMT2b* are the likely basis of sugar storage in the vacuoles of mature sugarcane stems. *ClTMT2* is mainly expressed in parenchymal cells of watermelon fruits. Ren et al. [13] obtained two *cltst2* mutants in watermelon using CRISPR/Cas9. The fruit sugar content decreased by about 35% at 30 dap and exhibited delayed rind coloration compared to WT, demonstrating the positive effect of ClTMT2 on the domestication of sugar accumulation in watermelon. TMT activity was positively correlated with seed yield, and *TMT* overexpression in cellular sugar-sensing machinery caused a decreased cytoplasmic glucose level. Increased expression of photosynthetic genes and *SUC2* following changes in cytosolic enzyme hexokinase1-sensing glucose levels causes enhanced CO_2_ fixation activity and phloem loading, and reduced CO_2_ release at night, which together coordinate the export of more nutrients from source leaves to seeds [124].

***Evolution and potential divergence of SP family members***. Gene duplication, including tandem and fragment duplication, is considered one of the major forces in the evolution and expansion of gene families [125]. Duplication events in the rice *SP* family were found only in the *STP*, *PLT*, and *ERD* subfamilies, with *STP* tandem gene clusters found in Chr1, Chr2, Chr4, and Chr7, while *ERD* tandem gene clusters are found in Chr3 and Chr5 [126]. The correlation coefficients between the *PLT* genes in rice and maize were mostly less than 0.8, and Pearson’s correlation coefficients of two and three *PLT* duplicated gene pairs were less than 0.8 within their genomes, respectively (*OsPLT11* and *12*, *OsPLT8* and *9*, *ZmPLT7* and *8*, *ZmPLT12* and *16*, and *ZmPLT4* and *6*), indicating that these duplicated gene pairs could be significantly sub-functionalized [118]. Klepek et al. [112] performed sequence alignment and internal duplication analysis of all PLT protein sequences in seven cereal crops and found that PLT protein sequences may originate from the internal duplication of the original six transmembrane helix units. *PLT* genes have a broad expression pattern, with *SbPLT3* predominantly expressed in the stem, *SbPLT6/8* highly expressed in both leaf and stem tissues [107], *OsPLT13* specifically expressed in roots, *OsPLT3/4* specifically expressed in pollen at different developmental stages [127], and *AtPLT1/2* are highly expressed in immature tissues such as germinating pollen and pollen tubes [112], suggesting the diversification of biological function in PLT.

***Biological functions of STP, pGlcT, ERDL/SFP proteins***. STPs are mostly found in sink tissues or symplastically isolated cells, such as pollen tubes, developing embryos, or guard cells [111,128]. AtSTP1 and AtSTP4 are major plasma membrane hexose transport proteins present in guard cells. Arabidopsis double mutant plants *stp1*:*stp4* showed significantly reduced glucose and fructose contents in guard cells under end-of-the-night (EoN) conditions, with almost undetectable glucose levels after 40 min of light, and impaired light-induced stomatal opening and stomatal closure in response to darkness, probably as a direct result of impaired glucose and/or fructose input [129]. STP is essential for the morphogenesis of organs such as pollen and seeds; for example, *AtSTP2*, *AtSTP6*, and *AtSTP11* exhibit pollen-specific expression patterns [111]. Moreover, *AtSTP9* is significantly expressed in germinating pollen tubes [111,117], and AtSTP9 can specifically transport glucose. OsMST5 is involved in rice pollen development [129], while OsMST8 is an anther-specific STP that is mainly expressed in tapetal cells early in pollen development [130,131]. Zhang et al. [132] isolated and identified a rice mutant of *carbon-starved anther* (*csa*), and the causal protein was a key transcriptional regulator of sugar allocation during male reproductive development, with reduced sugar and starch levels in floral organs and greatly reduced expression of *OsMST8* in *csa* anthers, with reduced carbohydrate content in anthers leading to male sterility. *OsSTP4* is a widely expressed gene, with high expression in sunken leaves, leaf sheaths, and embryos [126]. Moreover, *SbSTP3* transcripts are higher in leaves than in other tissues [107]. Knockout mutants of *AtSTP1* show significantly reduced glucose uptake in the root system, indicating that AtSTP1 is the main transporter of external hexose uptake in roots [111]. The expression pattern of *STP* correlates strongly with the rapid accumulation of hexose in fruit vacuoles, for instance, the RNAi-mediated synergistic inhibition of three tomato *STP*s (*LeHT1-3*) resulted in a 55% reduction in fruit hexose accumulation [133], and microarray analysis showed that the grape STP subfamily members *VvHT2*, *VvHT3*, and *VvHT11* were highly expressed during fruit set to ripening and play a role in extracting hexoses from vascular parenchyma isomers [134].

*SbERD3* is highly expressed in leaf and stem tissues [107]. AtSFP1/2 are stress-induced diffusion-promoting transporter proteins that differ greatly in spatial and temporal expression [72]. *SbpGlcT1* is highly expressed in leaves, stems, and seeds [107]. The Arabidopsis *pglct-1*: *mex1* (maltose transporter) double mutant exhibits severely reduced sucrose content, severely inhibited starch accumulation and degradation, and limited photosynthetic activity in leaves for the defective output of photo-assimilates by chloroplasts, indicating that pGlcT and MEX1 play important roles in the output of starch degradation products from the chloroplast in Arabidopsis leaves [130].

## 3. Concluding Remarks and Future Perspectives

Over the past 30 years, a large number of studies have led to the systematic identification and functional characterization of SWEET, SUT, and SP transporter members in Arabidopsis and many crop species (representative examples summarized in Table 1). Importantly, the substrate specificities/preferences and transportation directions of several transporter subfamilies have been extensively studied, generally demonstrating the cross-species conservation for different phylogenetic groups of the transporter families among species [20,21,22].

To summarize, advancements are being achieved from three aspects: (1) The discoveries about the biochemical and molecular functions of major sugar transporters tell us generally, which sugars can be transported by a given transporter, especially when such a transporter shares the same phylogenetic group/subgroup with functionally characterized transporters. The concordance between the phylogeny and the molecular function of a sugar transporter has helped with the inference of key transporters for crop improvements; for example, TMT1 and TMT2 are mainly responsible for the uptake of hexose and sucrose, respectively, from the cytoplasm to the vacuole in plant cells, and consistent with this functional knowledge, RNAi knockdown of the sugarcane *TMT1*, *TMT2a*, and *TMT2b* dramatically reduced the total sugar content in sugarcane stems (nearly 80%), validating the essential function of TMT in vacuolar sucrose accumulation as discussed previously for the stem of sugarcane and sweet sorghum [39,141,142]. (2) The advancements in protein structural studies of sugar transporters and AI-facilitated protein structure prediction help us to evaluate whether a sugar transporter (in a given accession of a crop species, once the sequence is available) could be functional or functionally impaired. A limited number of sugar transporters from the model plant species have been structurally resolved; nonetheless, these pieces of information provide a foundation for the AI-enabled accurate prediction of sugar transporters from other crops [46,50,53,143,144]. Recent studies have demonstrated that AI-enabled protein prediction and rational design can be applied to crop improvement. For example, Wang et al. leveraged AlphaFold and found that the C-terminal variants of GmSWEET10a conferred enhanced or reduced transport activity, guiding the gene editing for GmSWEET10a functional improvement and leading to increased oil content in soybean [14]. (3) The widespread use of multi-omics (including transcriptomics, proteomics, and phosphoproteomics) allows for the accumulation of time-series omics datasets focused on a given biological process (for example, sugar accumulation in the sweet sorghum stem and grain filling in maize kernels) at a relatively low cost with a fine resolution. In particular, single-cell omics and spatial transcriptomic technologies provide more powerful tools for analyzing gene expression at a single-cell resolution with spatial tissue/cell-type information [145,146,147,148,149].

**Figure 4:**
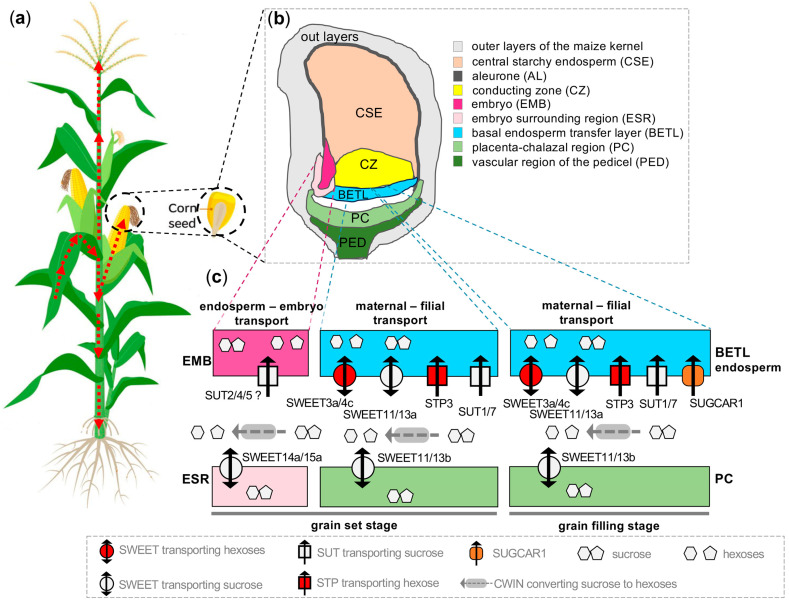
A schematic model of carbon allocation from “source” tissues to “sink” tissues using the maize plant as an illustration model and the representative molecular view of sugar transporter-mediated grain filling: (**a**) A schematic model of carbon allocation in maize plants. Red broken arrows indicate that the photosynthetically produced carbohydrates in mature leaves (the “source” tissue) are transported to other “sink” tissues, including the root, tassel, and corn cobs. (**b**) A graphic representation of an immature maize kernel showing different tissues, including the outer layers, central starchy endosperm (CSE), aleurone (AL), conducting zone (CZ), basal endosperm transfer layer (BETL), placento-chalazal region (PC), the vascular region of pedicel (PED), embryo (EMB), and the embryo surrounding region (ESR). The different tissues in maize kernels are color-coded and explained in the legend. This figure is adopted from previous studies [66,152]. (**c**) A hypothetical molecular view of the sugar transporter-mediated grain filling process. Different sugar transporters are dynamically involved in the sugar allocation from maternal to filial tissues in the grain. Sugar transporters with gene expression and genetic evidence are included in the model, with their sugar transporting directions illustrated. Apoplastic gaps exist between the maternal and filial tissues (the PC and BETL, respectively), and between the embryo-surrounding region (ESR) and embryo (EMB). SWEET, Sugar Will Eventually be Exported Transporters; SUT, sucrose transporter; STP, sugar transport protein; SUGCAR1, sucrose and glucose carrier 1; CWIN, cell wall invertase.

There have been several successful attempts to obtain a holistic view of sugar transportation in biological processes. Zhang et al. analyzed the spatiotemporal expression profiles of 105 sugar transporter-encoding genes in the leaves and stems of sugarcane relevant to the stem sugar accumulation in sugarcane, forming an expression pattern-guided view of the physiological functions of the SP family during phloem loading and unloading [141]. Ruan et al. integrated the dynamic expression patterns of genes encoding major sugar metabolic enzymes and transporters (i.e., the STP, SUT, and SWEET families), creating a model of the molecular control of sucrose unloading and partitioning within maize grains during grain filling [66]. Considering that the integration of different sugar transporters greatly enhances our understanding of the sugar transportation into maize kernels, we use this as a representative pathway to illustrate how a unifying figure can be achieved through such integration (Figure 4). In the maize plant, photo-assimilates are collected and transported via phloem from the source leaves to sink tissues, including developing seeds on the cob, developing tassels, and roots (Figure 4a). In the maize kernel, sucrose, as the major type of sugar being transported, is diffused from the placento-chalazal region (PC) into the apoplastic region (the maternal-filial interface), while sucrose and its catalyzed glucose and fructose are collected by several types of sugar transporters on the basal endosperm transfer layer (BETL) that is specialized for nutrient uptake. Moreover, the sugar exportation in the embryo-surrounding region (ESR) and collection by the embryo (EMB) reflect another important aspect of sugar transport between the endosperm and embryo (Figure 4b,c). Sucrose diffusion from the PC to the apoplasm is mainly mediated by SWEET11/13. In the apoplastic region, the high cell wall invertase activity ensures sucrose degradation into glucose and fructose to maintain the high-to-low sucrose gradient between the PC and the apoplast. On the other side of the apoplast, the basal endosperm transfer layer (BETL), specialized for nutrient uptake, harbors several types of sugar transporters, including STP3 and SWEET3a/c to collect glucose and SUT1/7 and SWEET11/13a to collect sucrose [66,150]. After the set stage, another atypical sugar transporter SUGCAR1 is highly expressed in the BETL to facilitate the collection of both sucrose and glucose during the grain filling stage [10,64,67]. In parallel, SWEET14a/15a mediates sugar exports from the ESR, and SUT2/4/5 are possibly responsible for sugar uptake supported at different grain developmental stages [66,150,151]. Extensive transcriptomics analyses via elaborately sampled RNA-seq, laser-capture micro-dissected (LCM) RNA-seq, and spatial transcriptomics contribute to the identification of the major transporters involved in the maize kernel development [66,150,152,153]. However, while we can now comprehend the role of sugar transporters in carbon allocation and grain development, knowledge of several sugar-transporter families, such as INT, pGlcT, PLT, SFP, STP, and VGT, remains scarce in cereal crops.

**Figure 5:**
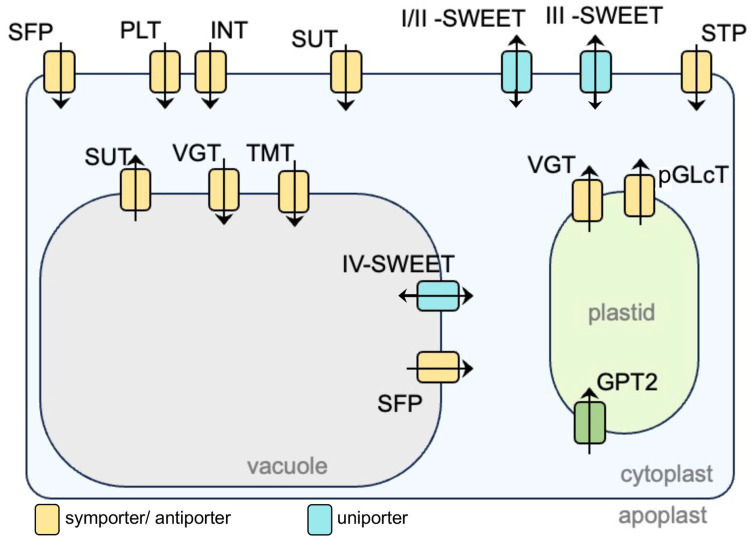
A proposed simplified model of different sugar transporters in a plant cell based on existing knowledge of their transported sugar molecules, transporting directions, and subcellular localizations. Arrowheads indicate hypothetical sugar-transporting directions. To focus on the major photosynthesis organelle, the plastid, and the major soluble sugar-storage organelle, the vacuole, other organelles are not shown in the figure. INT, inositol transporter; PLT, polyol transporter; pGLcT, plastidic glucose transporter; SFP, sugar facilitator protein; SUT, sucrose transporter; SWEET, Sugars Will Eventually Be Exported Transporter; TMT, tonoplast monosaccharide transporter; VGT, vacuolar glucose transporter. GPT2, glucose-6-phosphate translocator.

By interpreting the LCM RNA-seq results and the biochemical properties and the likely subcellular localizations of different sugar transporters reviewed in Arabidopsis, we propose a simplified hypothetical model for locating these sugar transporter proteins in a plant cell (not specifying the cell type and the source of tissues or organs; Figure 5) [21,152,154]. The major photosynthesis organelle, the plastid, and the major sugar-storage organelle, the vacuole, are illustrated in the figure, while other organelles have been omitted. The substrates can be monosaccharides, disaccharides, sugar alcohols, and polyols. Meanwhile, single-cell transcriptomic and spatiotemporal transcriptomic data sets have emerged for major crops (e.g., maize and wheat) focused on key developmental processes related to carbohydrate accumulation and yield, such as seed germination, embryo/endosperm development, and grain filling [155,156,157,158]. We believe that for a given biological context of crop development, this simplified model of sugar transporters would help to identify the major candidate genes encoding each of the sugar transporter families, as well as potentially contribute to the understanding of sugar import and export and the coordination between different transporters. In addition, by summarizing the established knowledge on different sugar transporters and introducing successful studies on the coordination between sugar transporters achieved in crops, we aim to point out several unaddressed questions that deserve immediate attention for future research. We exemplify some of these questions as follows. (1) How do plasma membrane-localized sugar transporters functionally coordinate with those localized on the tonoplast membrane? (2) Multiple sugar transporters from different families, including TMT, VGT, SUT, SWEET, and ERDL/SFP, are localized on the tonoplast. How do they functionally coordinate the import and export of different sugars? Do tonoplast membrane-localized transporters participate in cytosolic sugar homeostasis, which is known to be crucial for cellular functions? (3) While the biological functions of many sugar transporters have been characterized, the transcriptional and post-translational regulation of sugar transporters remains to be explored. Further advances in understanding the transcriptional and post-translational control of sugar transporters, as well as in manipulating these regulatory relationships, could help achieve tailored agronomic benefits in cereal crops.

In summary, integrating the above-mentioned three aspects of the advancements, together with new high-resolution RNA-seq data focusing on a given biological process in crops (for instance, soluble sugar accumulation in the stem of sweet sorghum and abiotic stress response in the leaf of wheat seedlings), will allow us to identify major sugar transporters, to comprehensively compare the expression changes in these transporters, and to generate a holistic view of sugar transportation during such a biological process. This will, in turn, enhance our understanding of crop physiology (particularly regarding the relationship between sugar partitioning and growth and development, biotic/abiotic tolerance and adaptation), generate testable hypotheses for crop genetic improvement, and highlight prioritized candidate sugar transporters for improving agronomic traits.

## Figures and Tables

**Table 1:** A summary of representative sugar transporters with functional characterization in plants.

Sugar Transport Protein	Monocot/Dicot	Protein *	Reported Substrate	Location	Reference
SWEET	Monocotyledons	ZmSWEET11/13a/14a/15a	Sucrose	Plasma membrane	[10,66,67]
	Monocotyledons	OsSWEET4/11/14/15	Sucrose	Plasma membrane	[40,43,62,63]
	Dicotyledons	AtSWEET1-3	2-Deoxyglucose	Plasma membrane/Tonoplast	[47,59,135]
	Dicotyledons	AtSWEET17	Fructose	Tonoplast	[135]
	Dicotyledons	AtSWEET9-15	Sucrose	Plasma membrane/Golgi apparatus	[58,61,136]
	Dicotyledons	AtSWEET4-8	Glucose	Plasma membrane/Tonoplast	[42,58]
	Monocotyledons	OsSWEET5	Galactose	Plasma membrane	[71]
	Dicotyledons	AtSWEET16	Fructose/Glucose/Sucrose	Tonoplast	[137]
SUT	Monocotyledons	ZmSUT2	Sucrose	Tonoplast	[20,100]
	Dicotyledons	AtSUC2/3	Sucrose	Plasma membrane	[78,80]
	Monocotyledons	OsSUT2/4	Sucrose	Tonoplast	[99,138]
	Monocotyledons	OsSUT1/3/5	Sucrose	Plasma membrane	[90]
	Monocotyledons	ZmSUT1	Sucrose	Plasma membrane	[76,93,94]
	Dicotyledons	AtSUC4	Sucrose	Plasma membrane/Tonoplast	[121,139]
SP	Dicotyledons	AtERD6	Glucose	Tonoplast	[72,113]
	Monocotyledons	OsTMT	Glucose	Tonoplast	[109]
	Dicotyledons	AtSTP1/2/3/4/6/11	Glucose/Mannose/Galactose/Xylose	Plasma membrane	[111]
	Dicotyledons	AtPLT5	Ribose/L-arose, polyols (Sorbitol, Xylitol/Erythritol/Oxocryl)	Plasma membrane	[112]
	Monocotyledons	OsSTP4/6	Glucose/Fructose/Galactose/Mannose	Plasma membrane	[114,115]
	Dicotyledons	AtINT1	Myo-inositol	Tonoplast	[118]
	Dicotyledons	AtINT2/4	Cyclic polyols	Plasma membrane	[118,140]
	Dicotyledons	AtSTP14	Galactose	Plasma membrane	[116]
	Dicotyledons	AtVGT1/2	Glucose	Tonoplast	[119]
	Dicotyledons	AtTMT1/2	Fructose/Glucose/Sucrose	Tonoplast	[120,121]
	Dicotyledons	AtSTP9	Glucose	Plasma membrane	[117]

*, the protein names include the Latin name abbreviations of their original species. At, *Arabidopsis thaliana*; Os, *Oryza sativa*; Zm, *Zea mays*.

## Data Availability

Data are contained within the article.

## Abbreviations

The following abbreviations are used in this manuscript:

SP	Sugar Porter
ST	Sugar Transporter
SUT	Sucrose Transporter
SWEET	Sugars Will Eventually Be Exported Transporters
STP	Sugar Transport Protein
TST	Tonoplast Sugar Transporter
TMT	Tonoplast Monosaccharide Transporter
VGT	Vacuolar Glucose Transporter
INT	Inositol Transporter
SFP	Sugar Facilitator Protein
pGlcT	Plastidic Glucose Transporter
PLT	Polyol Transporter
HT	Hexose Transporter
ERD	Early Responsive to Dehydration Protein
MST	Monosaccharide Transporter
MFS	Major Facilitator Superfamily
GPH	Glycoside–Pentoside–Hxuronide
SUC	Sucrose Transporter
RFO	Raffinose Family Oligosaccharides
AI	Artificial Intelligence
PP	Phloem Parenchyma
SE	Sieve Element
CC	Companion Cell
UDPG	Uridine Diphosphate Glucose
